# Interaction Information Along Lifespan of the Resting Brain Dynamics Reveals a Major Redundant Role of the Default Mode Network

**DOI:** 10.3390/e20100742

**Published:** 2018-09-28

**Authors:** Borja Camino-Pontes, Ibai Diez, Antonio Jimenez-Marin, Javier Rasero, Asier Erramuzpe, Paolo Bonifazi, Sebastiano Stramaglia, Stephan Swinnen, Jesus M. Cortes

**Affiliations:** 1 Computational Neuroimaging Lab, Biocruces Health Research Institute, 48903 Barakaldo, Spain; 2 Functional Neurology Research Group, Department of Neurology, Massachusetts General Hospital, Harvard Medical School, Boston, MA 02115, USA; 3 Gordon Center, Department of Nuclear Medicine, Massachusetts General Hospital, Harvard Medical School, Boston, MA 02115, USA; 4 Neurotechnology Laboratory, Tecnalia Health Department, 48160 Derio, Spain; 5 IKERBASQUE: The Basque Foundation for Science, 48013 Bilbao, Spain; 6 Dipartimento Interateneo di Fisica, Universita di Bari, and INFN, 70126 Bari, Italy; 7 Movement Control and Neuroplasticity Research Group, Department of Movement Sciences, KU Leuven, 3001 Leuven, Belgium; 8 Leuven Brain Institute (LBI), KU Leuven, 3000 Leuven, Belgium; 9 Department of Cell Biology and Histology, University of the Basque Country, 48940 Leioa, Spain

**Keywords:** interaction information, synergy, redundancy, default mode network, resting state, lifespan

## Abstract

Interaction Information (II) generalizes the univariate Shannon entropy to triplets of variables, allowing the detection of redundant (R) or synergetic (S) interactions in dynamical networks. Here, we calculated II from functional magnetic resonance imaging data and asked whether R or S vary across brain regions and along lifespan. Preserved along lifespan, we found high overlapping between the pattern of high R and the default mode network, whereas high values of S were overlapping with different cognitive domains, such as spatial and temporal memory, emotion processing and motor skills. Moreover, we have found a robust balance between R and S among different age intervals, indicating informational compensatory mechanisms in brain networks.

## 1. Introduction

The use of interaction information (II) can detect redundant or synergetic interactions in dynamical networks. Defined for a set of three variables, II measures the change in the mutual information between any two variables after adding the third. If the change is positive, a synergetic interaction occurs in the triplet, whereas if the change is negative, redundant interactions emerge [1,2]. Therefore, whilst the mutual information (MI) shared between two variables is always positive or zero (for the case of independent variables), II can be either positive or negative, respectively, unveiling redundancy (R) or synergy (S).

To give some specific examples, positive II (redundancy) results from common-cause structures when two variables share the same information about the third variable [3,4,5]. The prototypical example of negative II, i.e., synergy, is a set of three variables where one is the output of an XOR gate from the other two variables, which are independent random inputs [6,7]. In the latter case only the joint knowledge of the two inputs provides information about the target variable. It is also worth mentioning that II has been generalized to multiplets of an arbitrary number of variables by exploiting a suitable expansion of the MI [8] or in the scenario of Granger causality [9]; such an expansion has subsequently been generalized to lagged interactions in [10].

The presence of synergetic effects is well-known to occur in sociological and psychological modeling, where (very often) there are some variables that increase the prediction power on different ones [11]. On the other hand, redundancy have been addressed before in gene regulatory networks [12,13] and electrophysiological data in patients with epilepsy [2] or with deficit of consciousness [14], but, the pattern of triplet interactions in functional magnetic resonance imaging is not yet well-understood. By using a different methodology based on Granger causality influence, the authors in [15] found that R regions occurred mainly due to voxel-contiguity and inter-hemispheric symmetry, while S occurred mainly between non-homologous region pairs in contra-lateral hemispheres.

The development of novel imaging techniques and in particular, advances in magnetic resonance imaging (MRI), have enabled the reconstruction of functional brain networks, for example, by calculating correlations between blood oxygen-level dependent time series, the so-called functional MRI. Here, we focus on the resting state, i.e., when the subject’s brain is not involved in any goal-oriented task, which has been shown to have a correlation structure quite robust across different subjects [16,17]. Very important from a methodological side (also showing robustness), far away different methods, such as seed-based correlation analysis [18], independent component analysis [19] or partial least squares decomposition [20], have provided the same structure in the functional correlation, a modular organization of different resting state networks (RSN). Very striking for cognitive researchers, the different RSNs resemble the activation maps obtained when subjects perform specific tasks, such as for instance, auditory, visual, sensory-motor or executive control [21].

From a clinical point of view, the study of the resting state is crucial and has a tremendous impact and future, as subjects do not need to understand and memorize complex cognitive tasks to be performed, challenging for some of the most common brain disorders. So far, the functional connectivity patterns at rest have been shown to be altered in many different pathological conditions, such as deficit of consciousness [22,23,24,25], schizophrenia [26,27], epilepsy [28] and Alzheimer’s Disease [29,30,31,32,33].

Here, we assess synergetic and redundant interactions along lifespan, calculating II from functional MRI in a population of participants with an age range from 10 to 80 years. Previous studies have addressed variations of functional connectivity along lifespan, but as a far as we know, none of them made use of II before. It has been shown for instance that the functional connectivity generally decreases along lifespan, specifically between anterior and posterior brain regions [34,35]. It has been also shown that network modularity (a.k.a. network segregation) decreases as well [36], a mechanism supporting the loss of cognitive specialization with aging. However, when looking to structural connectivity rather than functional, not only network modularity decreases with age, but network integration increases [37], in a counterbalanced manner ensuring network efficiency along the lifespan. Therefore, despite previous work approaching functional connectivity variations along lifespan, the use of II from functional MRI to approach synergetic and redundant interactions along lifespan have not been addressed before.

## 2. Methodology

### 2.1. Participants

Participants were recruited in the vicinity of Leuven and Hasselt (Belgium) from the general population by advertisements on websites, announcements at meetings and provision of flyers at visits of organizations, and public gatherings (PI: Stephan Swinnen). A sample of N=164 healthy volunteers (81 females) ranging in age from 10 to 80 years (mean age 44.4 years, SD 22.1 years) participated in the study. All participants were right-handed, as verified by the Edinburgh Handedness Inventory. None of the participants had a history of ophthalmological, neurological, psychiatric, or cardiovascular diseases potentially influencing imaging or clinical measures. Informed consent was obtained before testing. The study was approved by the local ethics committee for biomedical research, and was performed in accordance with the Declaration of Helsinki.

### 2.2. Imaging Acquisition

Image acquisition was performed in an MRI Siemens 3T MAGNETOM Trio MRI scanner with a 12-channel matrix head coil. The anatomical data was acquired as a high-resolution T1 image with a 3D magnetization prepared rapid acquisition gradient echo: repetition time (RT) = 2300 ms, echo time (ET) = 2.98 ms, voxel size =1×1×1.1 ${\mathrm{mm}}^{3}$, slice thickness = 1.1 mm, field of view =256×240 ${\mathrm{mm}}^{2}$, 160 contiguous sagittal slices covering the entire brain and brainstem.

Resting state functional data was acquired with a gradient echo-planar imaging sequence over a 10 min session using the following parameters: 200 whole-brain volumes with TR/TE = 3000/30 ms, flip angle =90, inter-slice gap =0.28 mm, voxel size =2.5×3×2.5 ${\mathrm{mm}}^{3}$, 80×80 matrix, slice thickness =2.8 mm, 50 oblique axial slices, interleaved in descending order.

### 2.3. Imaging Preprocessing

We applied resting functional MRI preprocessing similar to previous work [38,39,40,41,42,43] using FSL and AFNI. First, slice-time was applied to the fMRI data set. Next, each volume was aligned to the middle volume to correct for head motion artifacts. After intensity normalization, we regressed out the motion time courses, the average cerebrospinal fluid (CSF) signal and the average white matter signal. Next, a band pass filter was applied between 0.01 and 0.08 Hz [44]. Next, the preprocessed functional data was spatially normalized to the MNI152 brain template, with a voxel size of 3 × 3 × 3 ${\mathrm{mm}}^{3}$. Next, all voxels were spatially smoothed with a 6 mm full width at half maximum isotropic Gaussian kernel. Finally, in addition to head motion correction, we performed scrubbing, by which time points with frame-wise displacements > 0.5 were interpolated by a cubic spline [45]. We further removed the effect of head motion using the global frame displacements as a noninterest covariate, as old participants moved more than the young (when representing the mean frame-wise displacement as a function of age provided a correlation value equal to 0.51 with *p*-value equal to $10^{-11}$), and this fact introduced nuisance correlations with age.

### 2.4. Brain Hierarchical Atlas

The brain was divided in 2514 brain regions that we grouped into modules using the brain hierarchical atlas (BHA), developed in [46] and applied by the authors in a traumatic injury study [47] and in a lifespan study [48]. The BHA is available to download at [49]. A new Phyton version that was developed during Brainhack Global 2017-Bilbao can be download at [50].

Although full details have been provided before [46], very briefly, the use of the BHA guarantees three conditions simultaneously: (1) that the dynamics of voxels belonging to a same module is very similar, (2) that those voxels within same module are structurally wired by white matter tracts, (3) that modules are simultaneously functional and structural.

Here, we focus on the M = 20 module partition as was shown to be optimal based on cross-modularity [46], and index defined as the geometric mean between the modularity of the structural partition, the modularity of the functional partition, and the mean Sorensen similarity between structural and functional modules.

**Figure 1:**
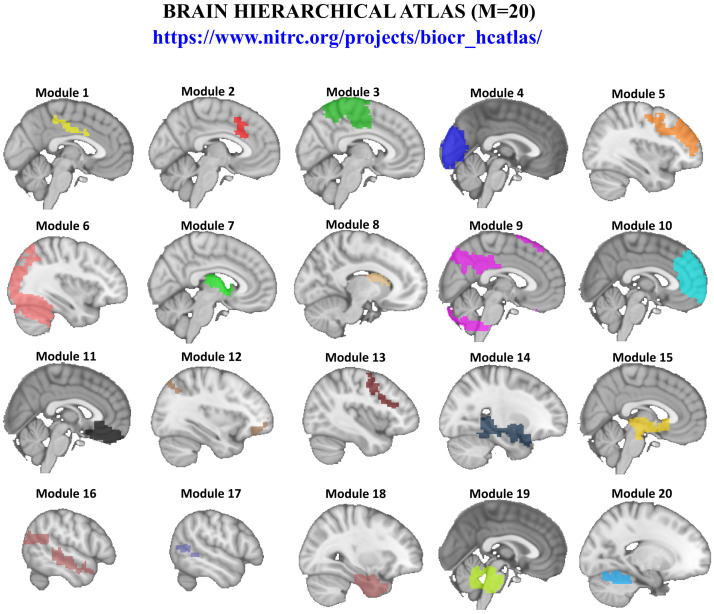
Brain Hierarchical Atlas (BHA). Available to download at [49], the BHA is used to define M = 20 modules, that was shown to be the optimal representation for best matching between brain structure and function. For each module, we only depict the sagittal slice that best represents the corresponding module. For a complete description of the BHA, see the Methodology section and reference [46].

A simple graph of the M = 20 modules is illustrated in Figure 1, but a full graphical representation (including axial, sagittal and coronal views) of the M = 20 modules can be found in Figures 3 and 4 in the paper [46], together with supplementary Figures S3 and S4 and supplementary videos (one per module) available at [46]. Moreover, a complete anatomical description for each module is provided in Table S1 in [46] and a functional correspondence for each module can be found in Figure 6b in [46].

### 2.5. Shannon Entropy

The Shannon entropy of a random variable *X* is defined as:

(1) $$\begin{array}{c} H\left(X\right)=-\sum_{x}\mathrm{prob}\left(x\right)\log\;\;\mathrm{prob}\left(x\right), \end{array}$$

where *x* represents one possible state of variable *X* [51,52]. Equation (1) can be generalized to two and three dimensions, respectively as $H\left(X,Y\right)=-\sum_{x}\sum_{y}\mathrm{prob}\left(x,y\right)\log\;\;\mathrm{prob}\left(x,y\right)$ and $H\left(X,Y,Z\right)=-\sum_{x}\sum_{y}\sum_{z}\mathrm{prob}\left(x,y,z\right)\log\;\;\mathrm{prob}\left(x,y,z\right)$. For base 2 logarithm (as we have done here), the entropy is expressed in bits.

Here, *X* represents any time series of resting state functional dynamics.

### 2.6. Interaction Information

The interaction information (II) is an extension of the Shannon entropy to triplets of variables [1]. For any triplet (X,Y,Z), the interaction information (II) is defined as

(2) $$\begin{array}{ccc} \mathrm{II}(X,Y,Z) & \equiv & \mathrm{MI}(X,Y)-\mathrm{MI}(X,Y|Z) \end{array}$$

where MI(X,Y)=H(X,Y)−H(X)−H(Y) is the mutual information between *X* and *Y* and MI(X,Y|Z) is the conditional mutual information between *X* and *Y* conditioned to *Z* (for details see [52]).

The sign of II has important physical implications; when II is positive, the three variables (X,Y,Z) are said to be redundant, while if II is negative, the interaction in (X,Y,Z) is synergetic.

Here, X,Y,Z represent any three time series of resting state functional dynamics.

### 2.7. Calculation of II

First, we made use of the BHA to define M = 20 modules (depicted in Figure 1), which maximizes the similarity between structural and functional modules (see above). Each module was used as a mask to extract the time series of the voxels belonging to it (in the 3 × 3 × 3 ${\mathrm{mm}}^{3}$ MNI template, on average, about 125 time series belong to a given module). Next, we built representative time series for each module by averaging over all the time series within it. Therefore, the brain dynamics was reduced to M = 20 time series, each per module.

For calculation of II, we used triplets (X,Y,Z) of module time series and applied Equations (2), estimating MI(X,Y) and MI(X,Y|Z) using the Gaussian copula approach recently derived in [53]. In particular, we made use of the functions *cmi_ggg.m* and *mi_gg.m*; available at [54].

Important to remark is that because the copula entropy does not depend on the marginal distributions of the original variables, the authors in [53] transformed the marginals to be standard Gaussian variables, and therefore, the MI was exactly calculated under the Gaussian assumption.

### 2.8. Per Module R and S

Values of R per brain module were obtained by summing (for a fixed module *m*) over all pairs such that II was positive, i.e.,

(3) $$\begin{array}{c} R_{m}\equiv \frac{1}{N^{+}}\sum_{y}\sum_{z}{\mathrm{II}}^{+}\left(X=m,Y,Z\right), \end{array}$$

where ${\mathrm{II}}^{+}$ represent any positive value of II and $N^{+}$ the total number of positive elements. Analogously, the per module S was defined as:

(4) $$\begin{array}{c} S_{m}\equiv \frac{1}{N^{-}}\sum_{y}\sum_{z}\left|{\mathrm{II}}^{-}\left(X=m,Y,Z\right)\right|, \end{array}$$

where ${\mathrm{II}}^{-}$ represent any negative value of II, $N^{-}$ the total number of negative elements and |⋯| absolute value.

For the calculation of both $R_{m}$ and $S_{m}$ we only considered triplets in which the three variables are distinct from each other, i.e., satisfying that y≠m, y≠z and z≠y.

Normalized values of R and S were calculated by dividing each value by its maximum.

### 2.9. Statistical Analysis

To study the effect of age on the variables R, S and the ratio R/S along lifespan, we divided the entire population of N=164 in 4 different intervals: I1 (10–20 years old, $N_{1}=30$), I2 (20–40 years old, $N_{2}=46$), I3 (40–60 years old, $N_{3}=29$) and I4 (60–80 years old, $N_{4}=59$).

Group comparison of variables R, S and the ratio R/S was performed following four stages: First, to eliminate statistical dependencies between values corresponding to different brain modules, for each participant we averaged over all brain modules R, S and R/S. Second, we performed a Kruskal-Wallis test between the values corresponding to the different age intervals. Third, we performed a Wilcoxon rank sum test as a post-hoc analysis between all pairs of comparisons. Fourth and last, we applied a Bonferroni correction by building a significance threshold equal to 0.05/6=0.0083 as we had four age intervals, and this provided six pairwise comparisons.

Validation of the Gaussian assumption for these variables R, S and the ratio R/S was assessed by a Shapiro-Wilk test together with a graphical manner such as a normal probability plot (shown in Figure S1).

All the statistical analyses were performed in MATLAB (version R2017a, MathWorks Inc., Natick, MA, USA).

### 2.10. Mask of the Resting State Networks

Following [55], we created a mask for the different resting state networks by defining voxels such that their *z*-score value satisfied z<−3 or z>3. In particular, we built masks for the default mode, cerebellum, executive control, frontoparietal, sensorimotor and visual resting state networks.

These masks were used to calculate the percentage of overlap between brain maps of R, S and R/S with the different functional resting state networks. Notice that, as these networks are overlapping each other, no normalization exist in these percentages.

## 3. Results

M = 20 modules of the BHA were used as regions of interest (Figure 1). We calculated II for all possible triplets. Redundancy and synergy were assessed using Equations (3) and (4). Lifespan was assessed defining four different intervals of age: I1 (10–20 years), I2 (20–40 years), I3 (40–60 years) and I4 (60–80 years).

**Figure 2:**
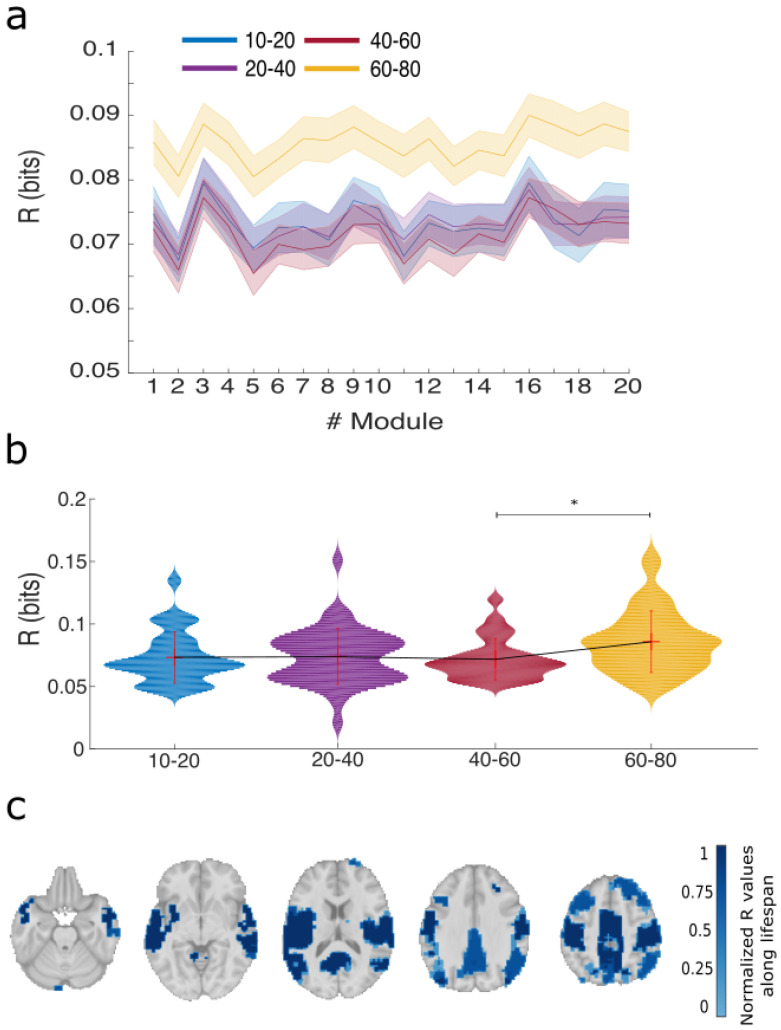
Variations of redundancy (R) per brain module and along lifespan. (**a**) For each module, values of R are represented in 4 different age intervals: blue (10–20 years old), purple (20–40), red (40–60) and magenta (60–80). Dark central lines represent average values across participants and shaded areas represent ± the standard error of the mean, calculated as the standard deviation of all values in the group divided by the square root of the group size; (**b**) Violin plots of R averaging over all brain modules within age interval. Mean ± standard deviation (no median) is also represented within each violin. The means of the different groups have been connected by a thin solid black line just to easily see the tendency of variations across age groups. * represents statistical significant differences after Bonferroni correction; (**c**) Brain maps of normalized R averaging over all age intervals with a threshold value of 0.7.

Values of R in bits are represented in Figure 2. Panel a shows the values of R per each of the M = 20 modules, at different age intervals. Along lifespan, the average R over all brain modules showed differences between groups (Kruskal-Wallis test, *p*-value of p=0.01). The non-parametric test was necessary as the average value of R over all brain modules was non-Gaussian for all age intervals (Figure S1). Post-hoc analyses (Figure 2b) between all pairs of groups revealed that only the comparison I3 vs. I4 was significant different after Bonferroni correction (Wilcoxon rank sum test, p=0.006).

Brain maps of normalized R values per module are represented in Figure 2c. Highest values were found in modules 3, 9 and 16, that bilaterally are located in cerebellum, precuneus, posterior cingulate, superior and middle temporal gyrus, paracentral lobule, precentral gyrus, superior frontal and parietal gyrus and insula. The function associated with these high redundant areas is a superposition of three important resting state networks, namely, default mode, sensory-motor and auditory networks.

**Figure 3:**
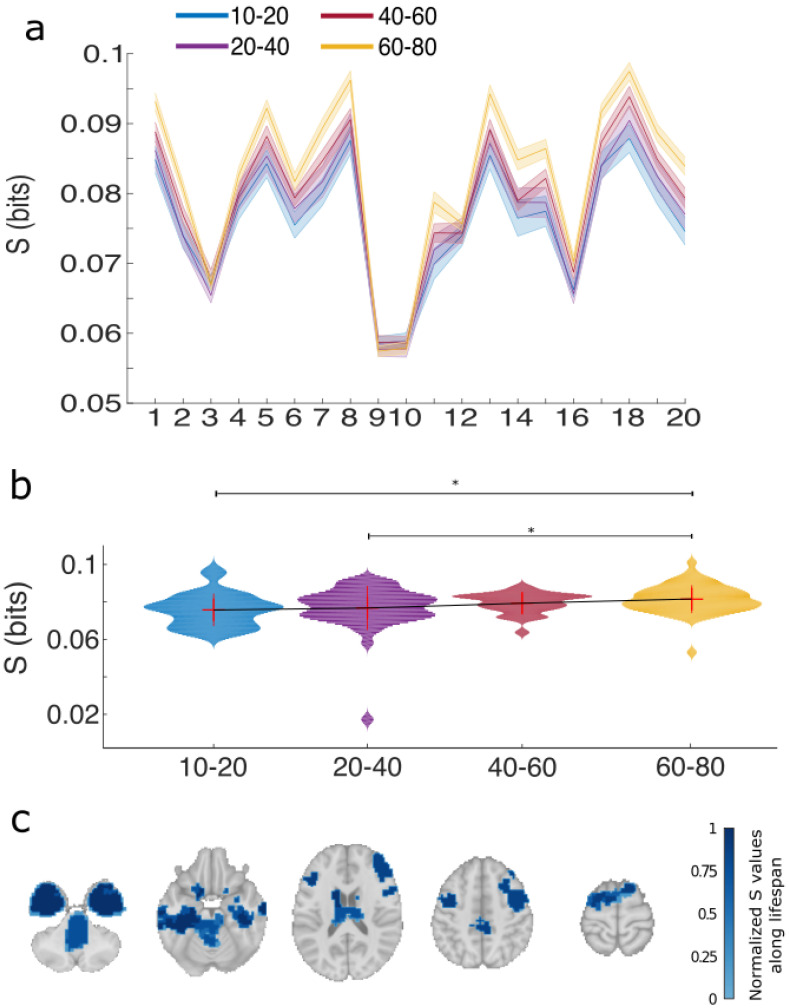
Variations of synergy (S) per brain module and along lifespan. (**a**) For each module, values of S are represented in 4 different age ranges, blue (10–20 years old), purple (20–40), red (40–60) and magenta (60–80). Dark central lines represent average values across participants and shaded areas represent ± the standard error of the mean, calculated as the standard deviation of all values in the group divided by the square root of the group size; (**b**) Violin plots of R averaging over all brain modules within age interval. Mean ± standard deviation (no median) is also represented within each violin. The means of the different groups have been connected by a thin solid black line just to easily see the tendency of variations across age groups. * represents statistical significant differences after Bonferroni correction; (**c**) Brain maps of normalized R averaging over all age intervals with a threshold value of 0.7.

Values of S in bits are represented in Figure 3. Panel a shows the values of S per each of the modules at different age intervals. Along lifespan, the average S over all brain modules showed differences between groups (Kruskal-Wallis test, p=0.002). Post-hoc analyses (Figure 3b) between all pairs of groups revealed that synergy was different for the comparisons I1 vs. I4 (Wilcoxon rank sum, p=0.0006) and I2 vs. I4 (Wilcoxon rank sum, p=0.006).

Brain maps of normalized S values per module are represented in Figure 3c. Highest values were found in modules 3, 8 and 18, that bilaterally are located in hippocampus, amygdala, entorhinal cortex, fusiform, temporal pole, inferior temporal gyrus, caudate and putamen. These areas are associated with different cognitive domains, such as spatial and temporal memory, emotion processing and motor skills.

**Figure 4:**
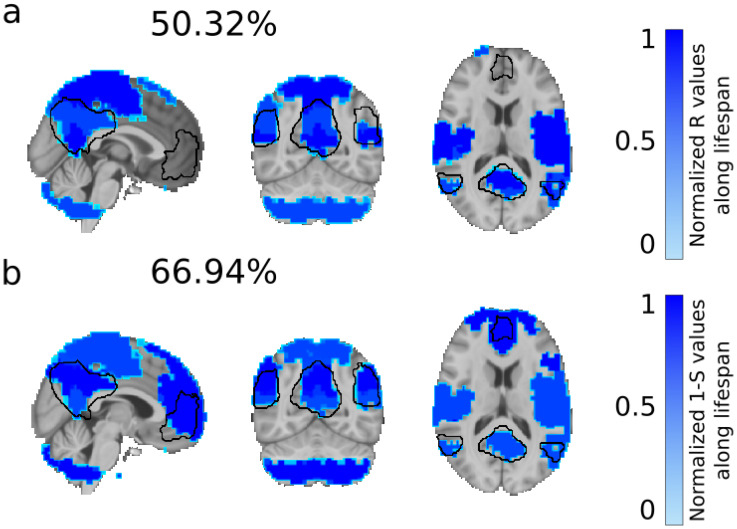
Normalized values of R and 1−S across brain regions reveals a key redundant role of the default mode network. (**a**) Brain maps of normalized R averaged over age intervals showed an overlap of 50.32% with the default mode network (depicted in black); (**b**) Plotting similar brain maps for 1−S increased the overlap with the default mode network up to 66.94%. Notice that 1−S but not R incorporated the frontal pole into the brain map, what caused to increase the matching with the default mode network.

Figure 4 shows brain maps of normalized R together with the ones for 1−S. Values with highest 1−S were found in modules 3, 9 and 10, bilaterally located in the anterior cingulate, inferior parietal and frontal gyrus, orbital gyrus, pars opercularis, pars orbitalis, pars triangularis, paracentral lobule, precentral gyrus, postcentral gyrus, precuneus, superior temporal gyrus, insula, cerebellum, posterior cingulate, inferior parietal gyrus, superior frontal gyrus. When comparing the brain maps of R and 1−S one can see how 1−S (but not R) incorporates the frontal pole, increasing the overlapping with the default mode network (DMN) from 50.32% for R (Figure 4a) to 66.95% for 1−S (Figure 4b).

**Figure 5:**
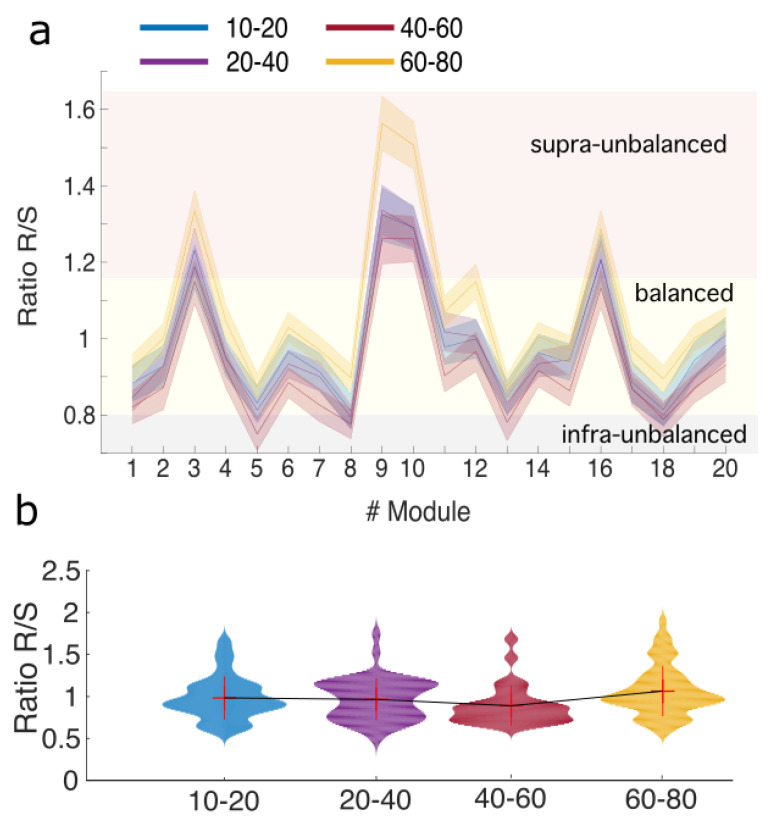
A balanced ratio R/S along lifespan suggests compensatory mechanisms between redundancy and synergy; (**a**) For each module, values of the ratio R/S are represented in 4 different age ranges, blue (10–20 years old), purple (20–40), red (40–60) and magenta (60–80). Three dashed lines delimit three regimes: 1. Infra-unbalanced, with values of R/S smaller than the mean minus one multiplied by the standard deviation (colored with light grey rectangle); 2. Supra-unbalanced, with values of R/S bigger than the mean plus one multiplied by the standard deviation (light red rectangle); and 3. Balanced, elsewhere (light yellow rectangle). Modules 9 and 10 corresponding to the default mode network are highly supra-unbalanced. Modules 5 and 8 corresponding to the fronto-parietal network are infra-unbalanced; (**b**) Violin plots of R/S averaging over all brain modules for each age interval. Mean ± standard deviation (no median) is also represented within each violin. The means of the different groups have been connected by a thin solid black line just to easily see the tendency of variations across age groups. No statistical differences occurred in any group comparison after Bonferroni correction, indicating a robust balance between R and S along lifespan.

We have found that the amount of R is somehow compensated by S, and this occurred for all brain modules and along lifespan (mean over all R/S values =0.98, standard deviation =0.16). This is illustrated in Figure 5a. Indeed, although both R and S showed differences along lifespan, the ratio R/S did not (Kruskal-Wallis test, p=0.08). Post-hoc pairwise comparisons did not show either any significant comparison (Figure 5b), indicating a robust balance between R and S along lifespan.

Despite the effective balance between R and S (captured by the ratio R/S close to 1), however, some brain areas went beyond the balanced regime, either to values of R much bigger or much smaller than S. To understand what brain regions correspond to each situation, we defined brain maps of infra-unbalanced R/S by looking to the brain areas with ratio R/S smaller than the mean minus one multiplied by the standard deviation. Similarly, supra-unbalanced brain maps were determined by looking to the ratio values bigger than the mean plus one multiplied by the standard deviation. Balanced areas corresponded to all the other situations of R/S. Table 1 shows the overlapping of the three classes of brain maps (infra-unbalanced, supra-unbalanced and balanced) with the most important resting state networks: default mode, cerebellum, executive control, frontoparietal, sensorimotor and visual. Very remarkably, infra-unbalanced brain maps overlapped 9.5% with the frontoparietal network. Balanced brain maps overlapped 84% and 77% respectively with the cerebellum and visual networks. Supra-unbalanced brain maps matched 69.18% with the DMN, again revealing a major redundant role of this network.

## 4. Discussion

Interaction information (II) allows to assess how information between pairs of variables is enhanced (by synergy, S) or diminished (by redundancy, R) after adding a third interacting variable. Here, using the brain’s oxygenation dynamics provided by functional MRI, we have studied how the values of R and S are distributed across brain areas and along lifespan.

Across brain areas, high values of S were found majorly in subcortical structures (amygdala, hippocampus, putamen and caudate), although some others were cortical (entorhinal cortex, fusiform and temporal pole), whilst R was found fully at the cerebral cortex (precuneus, posterior cingulate, superior and middle temporal gyrus, paracentral lobule, precentral gyrus, superior frontal and parietal gyrus and insula) and in the cerebellum. At the functional level, S was associated with spatial and temporal memory, emotion processing and motor skills, whereas R was associated with sensory processing (auditory and visual) and to a major extent to the DMN.

When looking to R and S across different age groups, we have found that the anatomical representation of R and S preserved along lifespan, although an increase in the magnitude of both R and S occurred for the group of participants older than 60 years as compared to younger populations.

This excess of R in the DMN occurring for the old population might be related with a network plasticity mechanism based on compensation, triggered after a brain insult and producing DMN hyperconnectivity occurring in the onset of several pathologies, for instance, after concussion [56,57] or in the early stage of Alzheimer’s disease [58].

The DMN has been shown to be altered in a plethora of brain disorders. Its redundant role found here, as yet unreported, might suggest the DMN to work as an information integrator at the large scale achieved by increasing redundancy. Perhaps, the significant increase of R occurring for the old population suggests a physiological dysfunction of the DMN when we age, known to be altered [59], but more research is needed to confirm this conjecture.

We have shown that the amount of R and S are roughly balanced (as the ratio R/S tends to 1) across brain areas and along lifespan, suggesting compensatory informational mechanisms in brain networks, that as far as we know, never before has been acknowledged. However, some specific networks go beyond the balanced regime, such as the frontoparietal network, which classically associated with attentional control [60] is the network most infra-unbalanced (i.e., with smaller values of the ratio R/S), revealing a new synergetic role of this network from an informational perspective. Moreover, cerebellum and visual are the two networks most balanced, similarly revealing new informational roles for these networks. Finally, the DMN is the one most supra-unbalanced (with the highest ratio R/S), and therefore, confirming from an information-compensation point of view, again, the redundant role of the DMN.

Future research should pay attention to what possible mechanisms or circuits can sustain R and S in the brain, for instance, addressing if some network topological metrics obtained from the structural connectivity matrix such as integration or segregation are somehow related to synergy and redundancy, although this is far the scope of the present work.

## Figures and Tables

**Table 1:** Overlapping percentage of the ratio R/S with the main Resting State Networks. Notice that the RSNs are overlapping networks each other, so overlapping percentage is not normalized.

	Infra-Balanced (%)	Balanced (%)	Supra-Balanced (%)
**Default Mode**	0.2246	19.0583	69.1893
**Auditory**	0.0284	38.1631	55.2979
**Cerebellum**	0	84.3334	0.8268
**Executive Control**	5.176	37.3482	30.9205
**Frontoparietal**	9.5896	39.358	39.5368
**Sensorimotor**	1.72	11.7076	57.8549
**Visual**	0	77.7678	5.9748

## Supplementary Materials

The following are available online at http://www.mdpi.com/1099-4300/20/10/742/s1, Figure S1: Validation of the Gaussian assumption for the different variables redundancy (R), synergy (S) and the ratio R/S.

## Abbreviations

II	interaction information
MI	mutual information
R	redundancy
S	synergy
MRI	magnetic resonance imaging
DMN	default mode network
RSN	resting state network
BHA	brain hierarchical atlas
