# Supplementary material for: Interaction Information Along Lifespan of the Resting Brain Dynamics Reveals a Major Redundant Role of the Default Mode Network
Source: Entropy (Basel). 2018 Sep 28;20(10):742. doi: 10.3390/e20100742 (PMC7512305; doi:10.3390/e20100742)
Supplement: Supplementary file 1 [file entropy-20-00742-s001.pdf]

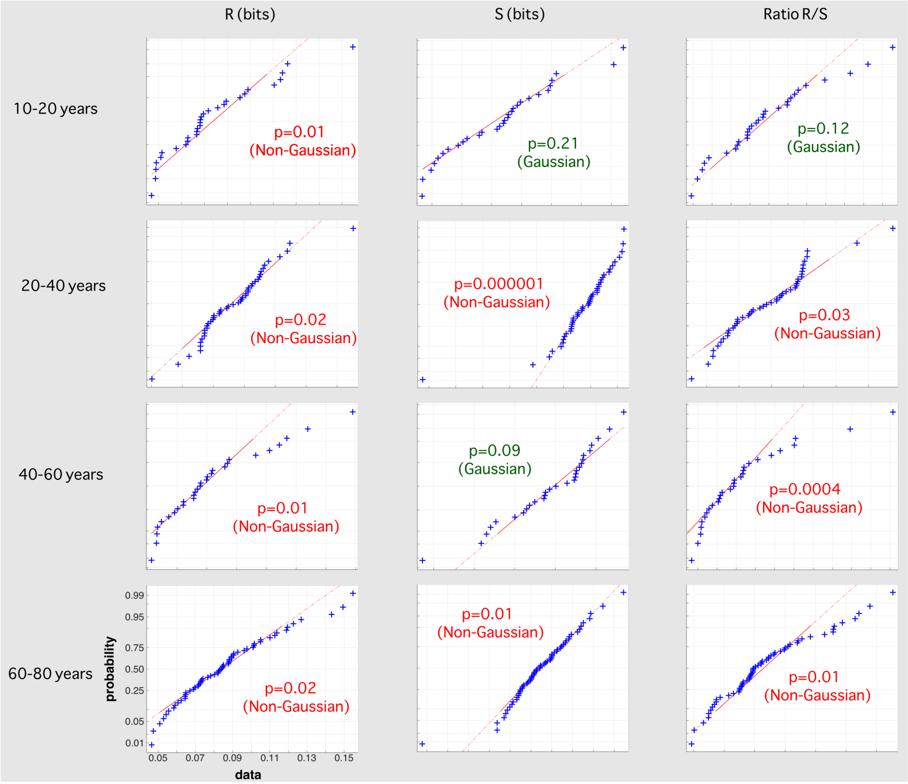

Figure S1. Validation of the Gaussian assumption for the different variables redundancy (R), synergy (S) and the ratio R/S. For the different age groups, values of R (left), S (middle) and R/S (right) are plotted using normal probability plots to graphically detect non-Gaussianity, i.e., when data probability points (in blue) deviate from the straight line (in red), the latter corresponding to the ideal situation of Gaussianity. P-values after Shapiro-Wilk tests are also provided. Gaussian and non-Gaussian situations were colored respectively in red and green for easy visualization.
